# Towards malaria risk prediction in Afghanistan using remote sensing

**DOI:** 10.1186/1475-2875-9-125

**Published:** 2010-05-13

**Authors:** Farida Adimi, Radina P Soebiyanto, Najibullah Safi, Richard Kiang

**Affiliations:** 1 Global Change Data Center, NASA Goddard Space Flight Center, Greenbelt, Maryland 20771, USA; 2 Wyle Information Systems, McLean, Virginia 22102, USA; 3 Goddard Earth Sciences and Technology Center, University of Maryland Baltimore County, Baltimore, Maryland 21228, USA; 4 National Malaria and Leishmaniasis Control Programme, Afghan Ministry of Public Health, Sanatorium Road, Darulaman, Kabul, Afghanistan

## Abstract

**Background:**

Malaria is a significant public health concern in Afghanistan. Currently, approximately 60% of the population, or nearly 14 million people, live in a malaria-endemic area. Afghanistan's diverse landscape and terrain contributes to the heterogeneous malaria prevalence across the country. Understanding the role of environmental variables on malaria transmission can further the effort for malaria control programme.

**Methods:**

Provincial malaria epidemiological data (2004-2007) collected by the health posts in 23 provinces were used in conjunction with space-borne observations from NASA satellites. Specifically, the environmental variables, including precipitation, temperature and vegetation index measured by the Tropical Rainfall Measuring Mission and the Moderate Resolution Imaging Spectoradiometer, were used. Regression techniques were employed to model malaria cases as a function of environmental predictors. The resulting model was used for predicting malaria risks in Afghanistan. The entire time series except the last 6 months is used for training, and the last 6-month data is used for prediction and validation.

**Results:**

Vegetation index, in general, is the strongest predictor, reflecting the fact that irrigation is the main factor that promotes malaria transmission in Afghanistan. Surface temperature is the second strongest predictor. Precipitation is not shown as a significant predictor, as it may not directly lead to higher larval population. Autoregressiveness of the malaria epidemiological data is apparent from the analysis. The malaria time series are modelled well, with provincial average R^2 ^of 0.845. Although the R^2 ^for prediction has larger variation, the total 6-month cases prediction is only 8.9% higher than the actual cases.

**Conclusions:**

The provincial monthly malaria cases can be modelled and predicted using satellite-measured environmental parameters with reasonable accuracy. The Third Strategic Approach of the WHO EMRO Malaria Control and Elimination Plan is aimed to develop a cost-effective surveillance system that includes forecasting, early warning and detection. The predictive and early warning capabilities shown in this paper support this strategy.

## Background

Malaria is a significant public health concern in Afghanistan. With military conflicts and instability that have lasted nearly three decades, the once successful malaria vertical control programme was long abandoned, and the public health infrastructure in Afghanistan has all but disappeared. Malaria outbreaks have recently re-emerged in rice growing Kundoz province between 2001-2005 as a result of returning refugees from neighbouring countries, intensified rice cultivation close to populated towns, and lack of any vector control measures [1]. In addition, during 2007-2009 malaria out breaks has been reported from Takhar and Badakhshan provinces [2]. It is only since 2004, through the collaborations among WHO, Afghan Ministry of Public Health, NGOs and international donors, that the efforts of rebuilding the public health infrastructure have begun.

Currently, approximately 60% of the population, or nearly 14 million people, live in endemic area. 414,407 malaria cases were reported in 2006 [3]. But WHO estimated that there could be as many as 600,000 cases per year [4]. Vivax malaria has been the most dominant infection in Afghanistan. Evidences from the 1990s showed that falciparum infection had increased. In the eastern region of the country, for example, falciparum infections previously accounted for only 1% of the total malaria infections, and in 1996 it increased to 20% [5]. The incidence of falciparum malaria then decreased significantly between 2002 and 2008. In 2007-2009, falciparum cases contribute to less than 5% of the malaria burden [2]. At present, falciparum cases have declined to the extent that the National Malaria and Leishmaniasis Control Programme (NMLCP) could not find even 50 cases per year at the sentinel site for drug efficacy study. For treating uncomplicated falciparum malaria and mixed vivax and falciparum infections, artesunate combination therapy (ACT) is the best option. Chloroquine remains the effective medicine for vivax malaria.

Because of the diversity in landscape and terrain of Afghanistan, malaria prevalence is heterogeneous - it is believed to be endemic in areas below 2,000 metres of elevation and highly prevalent in river valleys, where rice growing is common [5]. However, in October of 2000 an epidemic of falciparum malaria was reported in the remote valleys of Bamian, which was previously malaria-free due to its relatively high altitude (2,250-2,400 m). The increase in population movements leads to increased potential for malaria transmission particularly during the short summer season, rendering high altitude area susceptible for infection.

The goal of the WHO Eastern Mediterranean Regional Office (EMRO) regional malaria programme is to reduce the malaria burden to a level at which it is no longer a major cause of morbidity and mortality and a barrier to social and economic development [6]. Specifically, the programme aims to reduce malaria morbidity by 60% and malaria mortality by 90% by the year 2013. The continuing military conflicts in Afghanistan, however, may limit the access to malaria treatment and prevention. Hence it will be challenging to reach as well as to maintain such goals.

Although not a traditional practice, insecticide-treated bed nets (ITN) have been gradually accepted through extensive public awareness campaigns and mobile, subsidized sales. In order to attain universal coverage in high-risk population, free distribution of long-lasting insecticide-impregnated nets (LLINs) have been adopted in 2008. For some localities, more than 75% of the residents own bed nets as shown in the November 2008 malaria indicators survey [3]. As insecticide residual spraying (IRS) is difficult to implement in the current situation, broadening the ITN use is a viable approach for reducing malaria infections. The continued, consistent and proper use of ITNs, however, still requires sustained awareness campaigns. Since other infectious diseases have also taken tolls in the population, malaria is grouped with other health and disease programmes into a basic package of health services (BPHS). The BPHS consist of tuberculosis, HIV/AIDS, immunization, maternal and child health, and Integrated Management of Childhood Illness (IMCI) [6,7]. The four levels of health services supporting BPHS are: the health posts (HP), the basic health centres (BHC), the comprehensive health centres (CHC), and the district hospitals (DH).

Much of Afghanistan is arid or semi-arid, and agriculture depends on irrigation and snow-fed rivers. The most prevalent malaria vector species are *Anopheles stephensi, Anopheles culicifacies*, *Anopheles pulcherrimus *and *Anopheles superpictus *[1]. These species breed in river pools, river edges, and irrigated rice fields. Melt snow in the spring and rainfall in the summer provide additional larval habitats and enhance malaria transmission. The main transmission season is from June to November, and transmission in other months is negligible. Normally, vivax transmission peaks in July, and falciparum transmission peaks in October [8].

Some environmental parameters are known to have specific roles in promoting malaria transmissions [7,9-13]. For example, temperature, rainfall and humidity influence the propagation and survivorship of the malaria vectors. Rice fields, water bodies and other land cover types are associated with the larval habitats of certain malaria vector species. The development of the Plasmodium parasites, as well as the sporogonic cycle is also largely temperature-dependent. Because all these environmental parameters are remotely sensed by satellites, remote sensing and Geographic Information System (GIS) are now the essential tools for analysing, modelling and predicting malaria transmissions. Climate forecast that uses satellite data to predict climatic conditions weeks to months ahead has also become an important method for projecting disease risks into the future as demonstrated by Thomson *et al *[13]. Remote-sensing based malaria early warning is now routinely used for some regions in Africa [11,12,14], but for other malaria endemic regions, such technology is less developed. For example, similar studies are scarce in Asia and even rarer for Afghanistan.

The satellite data used in this study for modelling malaria incidence include precipitation measured by the Tropical Rainfall Measuring Mission (TRMM) as well as land surface temperature and vegetation index from the MODerate resolution Imaging Spectroradiometer (MODIS) [15,16]. Launched in late 1997, TRMM is a joint mission between NASA and the Japan Aerospace Exploration Agency (JAXA) designed to monitor and study tropical rainfall, as well as to improve our understanding of the water cycle in the climate system. Of the five instruments carried by TRMM, the Precipitation Radar (PR) and the TRMM Microwave Imager (TMI) are most directly related to rain measurements. TMI is a nine-channel passive microwave imager which can be used for observing tropical cyclones in near-real-time among other applications. PR is the first and only space-based rain radar and has the ability of mapping a three-dimensional structure for rainfall. The other three instruments on board TRMM are the Visible and InfraRed Scanner (VIRS), the Lightning Imaging Sensor (LIS), and the Cloud & Earth Radiant Energy System (CERES). Additional information concerning TRMM can be found at [15].

Land surface temperature and vegetation index are two of the many geophysical parameters measured by the Moderate Resolution Imaging Spectroradiometer (MODIS) aboard Terra spacecraft. The MODIS instrument has 2 bands at 250 metres resolution, five bands at 500 metres, and 29 bands at 1,000 metres, with its spectral region ranging from visible to thermal wavelengths. Both Terra (launched December 1999) and Aqua (launched April 2002) carry MODIS. MODIS provides a wide variety of geophysical parameters including: surface reflectance and temperature, land cover/change, vegetation indices, thermal anomalies, aerosols, cloud products, atmospheric profiles, snow and sea ice cover, and ocean products.

Although it is known that malaria occurs in regions below 2,000 m in Afghanistan, elevation will not be used as a predictor in this study. Because the resolution for elevation data needs to match the resolution of the epidemiological data, elevation at provincial resolution is not a meaningful measure. On the other hand, if location-specific malaria survey data is available, NASA's 90 m resolution Shuttle Radar Topography Mission data would be very useful.

The precise relationship between malaria transmission and the environmental parameters, however, is complex. For example, while precipitation creates potential larval habitats, too much precipitation in too short a period may wash larvae away. Likewise, droughts do away with larval habitats, but may also weaken or reduce the predator populations and result in more intense malaria transmission later on. Coupled with the influences from other environmental and contextual determinants, malaria transmission is a nonlinear phenomenon but may still be approximated with regressions using linear predictors. Previously, the neural network method, a non-linear technique, has been used to model the malaria transmission in Thailand [10] and other Southeast Asian countries, and the results are sufficiently accurate for operational use.

In this study, statistical techniques are used to model and predict malaria cases in Afghanistan. The specific objective is to demonstrate that an early warning capability can be developed with such techniques using satellite-measured meteorological and environmental variables, such that the public health organizations can more effectively respond to malaria-related problems.

## Methods

### Study area

Afghanistan is a landlocked country in south central Asia (33 00 N, 65 00 E). Its climate is arid to semiarid with frequent sand storms. The northern and south-eastern areas of the country have very rugged mountain terrains (Hindu Kush), while the western and southern parts are flat deserts and plains. Except for some fertile valleys, the country has sparse vegetation and very few large permanent rivers. As a result, only about 12% of the land area is considered arable, of which less than 1% provides any permanent crops. Climate varies according to elevation and location. For example, Kabul (1,795 m) has cold winters and pleasant summers; Jalalabad (550 m) is subtropical; and Kandahar (1,006 m) is mild all year. Temperature also has wide variations; it may range from freezing at dawn to nearly 38°C at noontime. In the northern valleys, temperature can reach 49°C in the summer. In the Hindu Kush, temperature can be as low as -20°C to -30°C in the winter. In general, malaria occurs at altitude below 2,000 m and is most prevalent in snow-fed river valleys and rice growing areas.

### Malaria surveillance data

23 provinces with more complete malaria time series were used in this study, as often it is more difficult to draw statistical conclusions from short or incomplete epidemiological time series. The malaria epidemiology data used in this study were collected by the community health posts in Afghanistan. Data from the BHCs, CHCs and DHs were not included. The health posts are part of the primary health care facilities; they are community based health services at the village level in Afghanistan. These health posts are operated by community health workers from their own homes, and they deliver limited services such as treating common illnesses, prenatal care, family planning and immunization. Among other duties, the health posts provide curative care for the community which includes the clinical diagnosis and treatment of uncomplicated malaria [7].

**Figure 1:**
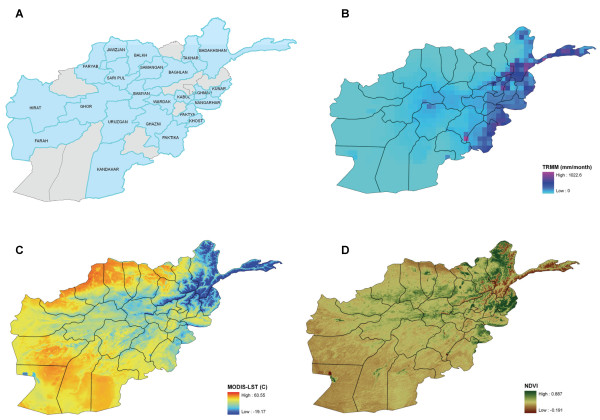
**Afghanistan map and environmental data for July 2005**. (A) Map of the 23 provinces in Afghanistan used in this study. (B) Total precipitation as measured by TRMM. (C) Average Land Surface Temperature (LST). (D) Average Normalized Difference Vegetation Index (NDVI).

The malaria data used in this study spans 2004-2007. Naturally, surveillance improved as the number of health posts grew, and may have contributed to an apparent increase in malaria cases or incidence. The malaria data used for this study is based on passive case detection and does not distinguish between the different parasites species. Figure 1 shows a map of the 23 provinces in Afghanistan that are used in this study.

### Climate and environmental data

#### Precipitation data

The monthly rainfall (Figure 1B) data used in modelling monthly malaria cases in Afghanistan was derived from the instruments on board the TRMM as previously described [16]. The TRMM precipitation data has a resolution of approximately 5 km at nadir. For this study, the monthly TRMM and other data sources rainfall estimate product (3B43 Version 6) [17] were used. This data set combines the microwave IR measurement and gauge adjustment at an almost global (between 50N and 50S degrees in latitude) fine resolution of 0.25 deg. The TRMM data set was acquired using the Giovanni system in the NASA's Goddard Earth Sciences (GES) Data and Information Services Center (DISC) [18].

#### Temperature and vegetation data

The land surface temperature (LST) and the normalized difference vegetation index (NDVI) are the two parameters used for this study and they are illustrated in Figure 1C-D. These datasets are distributed by the Land Processing Distributed Active Archive Center (LPDAAC) of the U.S. Geological Survey (USGS). Both are at 1 km spatial resolution with a sinusoidal projection; the temporal resolution for the LST data set is 8 days, while the NDVI data set is at a monthly temporal resolution. As the malaria time series is at a monthly resolution, the LST data are aggregated to a monthly resolution. In addition, both parameters are re-projected to a geographical projection compatible with the other datasets used in this study.

#### Statistical modelling

Linear regression is used to model the dependency of the malaria cases on the environmental parameters. The monthly provincial malaria cases are expressed as the weighted sum of the environmental parameters EV, the autoregressive terms AR, the seasonal variation S, and the trend *f*. Specifically,

Where,

*f(t) *accounts for the general trend owing to factors other than environmental in nature, such as improvement in public health support or population movements.

The regression model is thus:

Where β_j _is the weight for the predictor X_j _that was selected using stepwise regression.

Data collected by Health Posts in 23 provinces spanning the period between 2004 and 2007 were used. The time series data is scarce for some provinces and may cover only two years or less of monthly malaria cases. The last six months of the data were reserved for hind-casting or validation. Therefore, the training period is from the beginning of the malaria time series to June 2007, and the prediction period is the last six months from July to December 2007. Both forward and backward selections were used for selecting the set of the appropriate predictors.

## Results

Results from the stepwise regression are summarized in Table 1 for the 23 provinces in Afghanistan included in this study. This table shows the weights for each predictor and the associated p-values. For this study, the level of significance in p-value used in the stepwise regression model was limited so that only environmental predictors with a p-value level less than 0.05 are included. The square root of the mean square errors are also listed in the last two columns of Table 1 for both the training and the prediction of the time series. The prediction portion of the time series constitutes of the last six months of the available monthly malaria cases in each province.

**Table 1:** Stepwise regression coefficients and p-value (in italic) for 23 provinces.

Province	Mos. of Data	Trend	Sine	Cosine	**AR**_**t-1**_	Other Selected Variables	Est. Coeff. *(p-value)*	Fit R^2^	Pred. R^2^	Fit RMSE	Pred. RMSE
**Badakhshan**	38	233.86 *(0.013)*	586.779 *(0.2066)*	768.54 *(0.044)*	-0.289 *(0.099)*	T_t_	56.453 *(0.0088)*	0.83	0.74	249.31	521.30
						NDVI_t-1_	6832.8 *(0.0375)*				
**Baghlan**	33		74.1832 *(0.3396)*	-131.4 *(8E-04)*	0.591 *(0.001)*	NDVI_t-3_	1987.3 *(0.0279)*	0.73	0.30	111.68	157.59
**Balkh**	39	95.029 *(0.004)*	-24.825 *(0.3368)*	49.655 *(0.037)*	0.261 *(0.166)*			0.71	0.04	82.58	157.15
**Bamyan**	36		215.355 *(0.0002)*	170.47 *(0.002)*	0.442 *(0.003)*	T_t_	8.8078 *(0.0007)*	0.72	0.31	36.67	98.30
						NDVI_t-1_	901.67 *(0.0011)*				
**Farah**	32	-23.88 *(0.042)*	-15.517 *(0.202)*	-11.69 *(0.28)*	0.375 *(0.045)*	P_t-3_	0.7921 *(0.018)*	0.73	0.72	25.89	24.56
**Faryab**	38	144.49 *(0.027)*	-244.84 *(0.001)*	-223.7 *(0.003)*	0.359 *(0.036)*	NDVI_t-3_	-2003.7 *(0.0094)*	0.79	0.03	148.07	453.55
**Ghazni**	34		114.143 *(0.085)*	-236.1 *(1E-04)*	0.077 *(0.557)*	AR_t-3_	0.884 *(1E-06)*	0.80	0.95	67.05	148.21
						T_t-3_	9.285 *(0.008)*				
**Ghor**	33		-81.766 *(0.0008)*	-57.95 *(0.001)*	0.111 *(0.597)*	NDVI_t_	-649.3 *(0.008)*	0.77	0.67	30.00	110.12
**Hirat**	41		287.797 *(0.0675)*	21.191 *(0.84)*	0.62 *(6E-08)*	T_t-1_	16.72 *(0.0495)*	0.88	0.95	142.04	114.05
						NDVI_t-3_	5785.3 *(0.0045)*				
**Jawzjan**	28		-20.164 *(0.6149)*	-115.9 *(0.025)*	0.121 *(0.654)*	AR_t-2_	1.179 *(0.015)*	0.64	0.18	110.03	296.63
**Kabul**	33	114.95 *(0.004)*	79.3475 *(0.2133)*	-141 *(0.002)*	0.368 *(0.066)*	T_t-3_	7.22 *(0.036)*	0.86	0.74	52.59	177.14
**Kandahar**	41		-61.739 *(0.0246)*	-50.58 *(0.119)*	0.838 *(2E-13)*	NDVI_t-3_	3377.7 *(0.0153)*	0.89	0.003	96.69	182.35
**Khost**	29	52.91 *(0.01)*	-121.83 *(2E-05)*	31.113 *(0.133)*	-0.108 *(0.555)*	AR_t-3_	-0.485 *(0.002)*	0.85	0.85	32.73	66.68
**Kunar**	20		-591.44 *(0.0929)*	-273.9 *(0.244)*	0.685 *(1E-03)*			0.87	0.07	486.76	1227.8
**Laghman**	21	546.22 *(0.014)*	-336.92 *(0.0138)*	-227.3 *(0.01)*	-0.431 *(0.16)*			0.64	0.03	153.47	527.21
**Nangarhar**	17		-37.264 *(0.7778)*	-226.9 *(0.03)*	0.68 *(0.037)*	AR_t-3_	0.692 *(0.04)*	0.86	0.02	101.51	892.51
**Paktika**	31	80.561 *(2E-04)*	95.2655 *(0.0059)*	-9.201 *(0.431)*	0.912 *(2E-06)*	AR_t-3_	-0.443 *(0.005)*	0.94	0.17	24.42	314.76
						T_t-2_	5.677 *(0.005)*				
						NDVI_t-2_	1421.04 *(0.0714)*				
**Paktya**	28		-100.69 *(0.0078)*	-42.4 *(0.047)*	0.28 *(0.215)*			0.76	0.74	55.24	115.36
**Samangan**	36		-2.1022 *(0.9324)*	-30.82 *(0.141)*	0.656 *(3E-04)*			0.53	0.01	71.57	39.52
**Sari Pul**	27		-26.792 *(0.2226)*	28.848 *(0.267)*	1.018 *(4E-04)*	NDVI_t-4_	-1004.8 *(0.01516)*	0.77	0.13	36.11	90.42
**Takhar**	37		918.364 *(0.0137)*	955.91 *(0.077)*	0.733 *(8E-08)*	T_t_	64.835 *(0.0151)*	0.92	0.95	340.25	239.61
						NDVI_t-1_	8005.4 *(0.0017)*				
**Uruzgan**	41		-5.1359 *(0.5149)*	-0.764 *(0.923)*	0.219 *(0.218)*			0.07	0.43	30.72	44.06
**Wardak**	30		-17.142 *(0.0022)*	-4.778 *(0.272)*	-0.421 *(0.048)*			0.42	0.78	13.31	5.69

Abbreviations: AR = Auto Regression term (cases); T = temperature; NDVI = Normalized Difference Vegetation Index; P = precipitation; Pred = Predicted; RMSE = Root Mean Squared Error.

**Figure 2:**
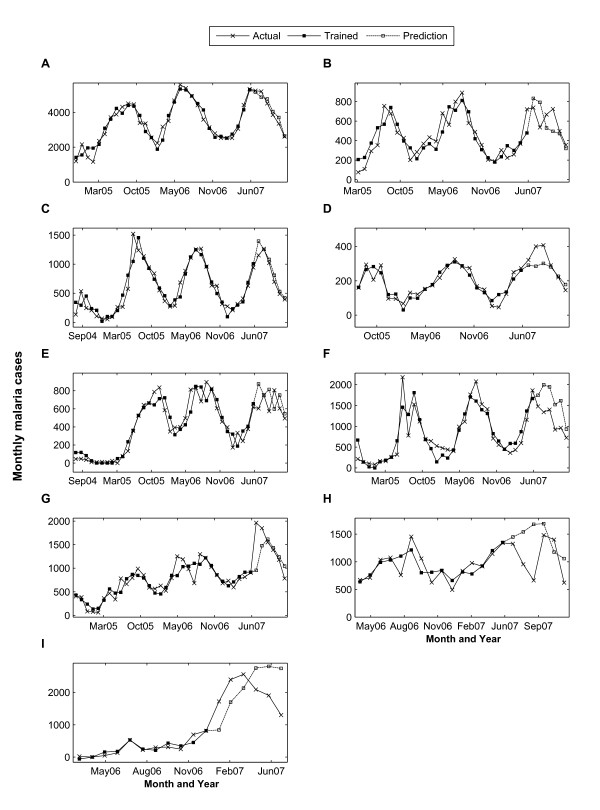
**Training and Prediction Results**. (A) Takhar, (B) Baghlan, (C) Hirat, (D) Khost, (E) Kandahar, (F) Badakhshan, (G) Faryab, (H) Laghman and (I) Nangarhar Province.

Seven of the 23 provinces (Badakhshan, Balkh, Faryab, Kabul, Khost, Laghman and Paktika) show a significant positive trend indicating rising malaria incidence during the training period (2004 to June 2007). The trend coefficient for Farah province on the other hand, is negative, indicating a significant decrease in malaria incidence during this period. The other 15 provinces do not show a consistent trend associated with malaria incidence during their training period. The training and prediction for the other 9 provinces are shown in Figure 2. The time series of the environmental variables for these 9 provinces are shown in Additional File 1.

Since malaria is an infectious disease, the malaria incidence for any given month will be autoregressive and show strong dependency on the previous month's infections. As shown in Table 1, thirteen of the provinces show significant contribution from the first order auto regression term (one month lag), while five provinces indicate more significant contribution from the second (two month lag) or third order (three month lag) auto regression terms.

The environmental parameters from NASA satellites such as temperature, vegetation, and precipitation were used as predictors for malaria risks in Afghanistan. Thirteen of the 23 provinces exhibit more significant contribution from at least one of the environmental determinants. Vegetation index appears to be one of the more significant predictors for malaria incidence in Afghanistan. Nine provinces (Badakhshan, Baghlan, Bamyan, Faryab, Ghor, Hirat, Kandahar, Sari Pul, and Takhar) have a p-value less than 0.05 for NDVI coefficients. The temperature factor exhibits a more significant contribution to malaria risk where seven of the 23 provinces under study show positive correlation between the average monthly temperature and malaria incidence. The ten provinces (Balkh, Jawzjan, Khost, Kunar, Laghman, Nangarhar, Paktya, Samangan, Uruzgan, and Wardak) which do not show appreciable relation from the environmental determinants all seem to have either a short time series data or sporadic malaria data.

As expected, precipitation in Afghanistan does not appear to be a significant factor for increased malaria risk. Because malaria risk is mainly due to irrigation, the vegetation index, which is associated with irrigation, is a strong predictor for transmission risks. The average R^2 ^weighed by malaria cases is 0.845.

**Figure 3:**
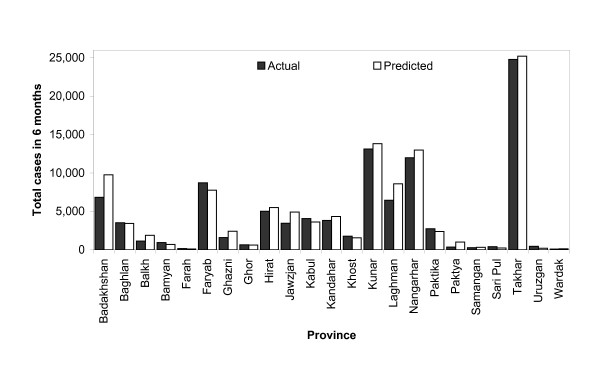
**Predicted and actual malaria cases in 6 months (July-December 2007) for 23 provinces**.

The last six months of the malaria time series for each province was reserved for hind-casting and validation. In this case, the trained weights were applied to the environmental, seasonal, trend, and autoregressive terms in the hindcast period. Since the monthly malaria cases are available during this period, the hindcasting method helps validate the model as well as test the prediction accuracy. This method was applied to 23 provinces in Afghanistan. The Root Mean Square Errors (RMSE) between the forecasted data and the actual monthly malaria cases for the July to December 2007 period are listed in Table 1 for all 23 provinces. In general, the RMSE from the hindcast period is larger than that of the fitted regions, as was expected since by design the training weights are deduced by minimizing the error between the fitted and the actual training data. Even though the RMSE is large for the forecasted period, the prediction tends to follow the trend of the real data. As shown in Table 1, the R^2 ^for prediction shows larger variation compared to the fitted dataset. Ignoring the three provinces with malaria time series shorter than 24 months, 10 provinces has R^2 ^> 0.5, and the other 10 provinces have R^2 ^< 0.5. Figure 3 illustrates the comparison between the actual malaria cases as reported by the health posts and the predicted cases from the current model for the 23 provinces. It shows an excellent agreement between the results from the regression model and the actual malaria incidence. The total number of predicted cases exceeds the actual cases only by 8.9%.

## Discussion

Meteorological and environmental parameters are the two types of variables that most directly affect malaria transmission. A number of other factors are also at play in influencing malaria epidemiology. For example, socioeconomic condition, public health service, military conflict and refugee movement may all modulate the suitability for malaria transmission. When these factors do not change suddenly (hence mathematically well-behaved), it is possible to use statistical techniques, like what have been shown in this study, to model the environmental effects on malaria transmission and to predict malaria incidence based on environmental parameters.

The continued military conflicts in Afghanistan may hinder the efforts for better malaria control and improving public health service. Such conflicts may result in damaged dwelling, creation of new mosquito breeding sites, living at unsuitable locations, lack of means for personal protection, difficulty of access to health care, movement of populations that may be infected with or have no immunity to malaria. The unpredictability of military conflicts may have contributed to the smaller R^2 ^in the prediction period for some of the provinces.

The regression models in this study include meteorological and environmental factors explicitly. The effects due to socioeconomic factors and military conflicts can be partially accounted for by the trend term. If these effects averaged over a province do not cause a sudden change in malaria transmission, then they can be accounted for by the first order trend term in the regression model. Second-order trend was also examined in the analysis but was found to be insignificant. On the other hand, improved socioeconomic conditions and reduced military conflicts would see a decrease of malaria transmission. In the 23 provinces studied, seven provinces show positive trend, one province show negative trend, and 15 provinces do not show any significant trend. Because the rebuilding of the public health infrastructure was started in 2004, the positive trend shown for these seven provinces could be due to improved surveillance instead of more intense transmission.

Short malaria time series pose a challenge for modelling and prediction. As shown in Figures 2H and 2I, when malaria time series are shorter than two years, it is difficult to capture the dependency on the remotely sensed parameters and project into the future.

In general, the regression model in this study can represent and predict malaria cases with reasonable accuracy when there is sufficient malaria epidemiological data available for modelling. In the prediction period, although there may be deviations between the predicted and the actual cases from month to month, the difference between the six-month sums is relatively small. This implies that public health organizations can use the six-month predictions to develop malaria response and control strategy at the appropriate level.

Closer inspection of Figure 3 shows that the hindcast monthly malaria cases are slightly higher than the actual cases reported. This over-estimation implies that the actual incidence is less than what is warranted by the environmental factors. This may be an indication that the concerted effort of malaria control in Afghanistan is bearing fruits - the overall malaria transmission in these 23 provinces is decreasing under largely the same environmental condition.

The third Strategic Approach of the WHO EMRO Malaria Control and Elimination Plan [6] is aimed to develop a cost-effective surveillance system that includes forecasting, early warning and detection that would lead to either the very early recognition of epidemics and immediate implementation of control measures or the implementation of preventive control measures before the epidemics starts. The predictive capabilities demonstrated in this study precisely support this strategy.

## Conclusions

This study shows that predicting malaria incidence using remotely sensed geophysical parameters is an achievable goal even with relatively limited epidemiological data and public health surveillance capability. For Afghanistan, a country with limited resources in an insecure environment, malaria forecasts and early warning system can help allocate public health resources in a most efficient way. As the conflicts and instability in Afghanistan may continue in the foreseeable future, it takes on a new urgency to implement such risk prediction capabilities for malaria control.

## Competing interests

The authors declare that they have no competing interests.

## Authors' contributions

FA and RK designed the study, analysed the data, and participated in drafting the manuscript; FA also participated in processing satellite data; RS participated in the analysis and drafting the manuscript; NS provided epidemiological data and information concerning the Afghan National Malaria and Leishmaniasis Control Programme, and participated in drafting the manuscript. The authors read and approved the manuscript.

## Supplementary Material

Additional file 1 **Monthly meteorological and environmental variables**. Monthly land surface temperature, NDVI, and TRMM-measured precipitation for 9 provinces. (A) Takhar, (B) Baghlan, (C) Hirat, (D) Khost, (E) Kandahar, (F) Badakhshan, (G) Faryab, (H) Laghman, (I) Nangarhar Province Click here for file
