# Supplementary material for: Towards malaria risk prediction in Afghanistan using remote sensing
Source: Malar J. 2010 May 13;9:125. doi: 10.1186/1475-2875-9-125 (PMC2878304; doi:10.1186/1475-2875-9-125)
Supplement: Additional file 1 — Monthly meteorological and environmental variables. Monthly land surface temperature, NDVI, and TRMM-measured precipitation for 9 provinces. (A) Takhar, (B) Baghlan, (C) Hirat, (D) Khost, (E) Kandahar, (F) Badakhshan, (G) Faryab, (H) Laghman, (I) Nangarhar Province [file 1475-2875-9-125-S1.PDF]

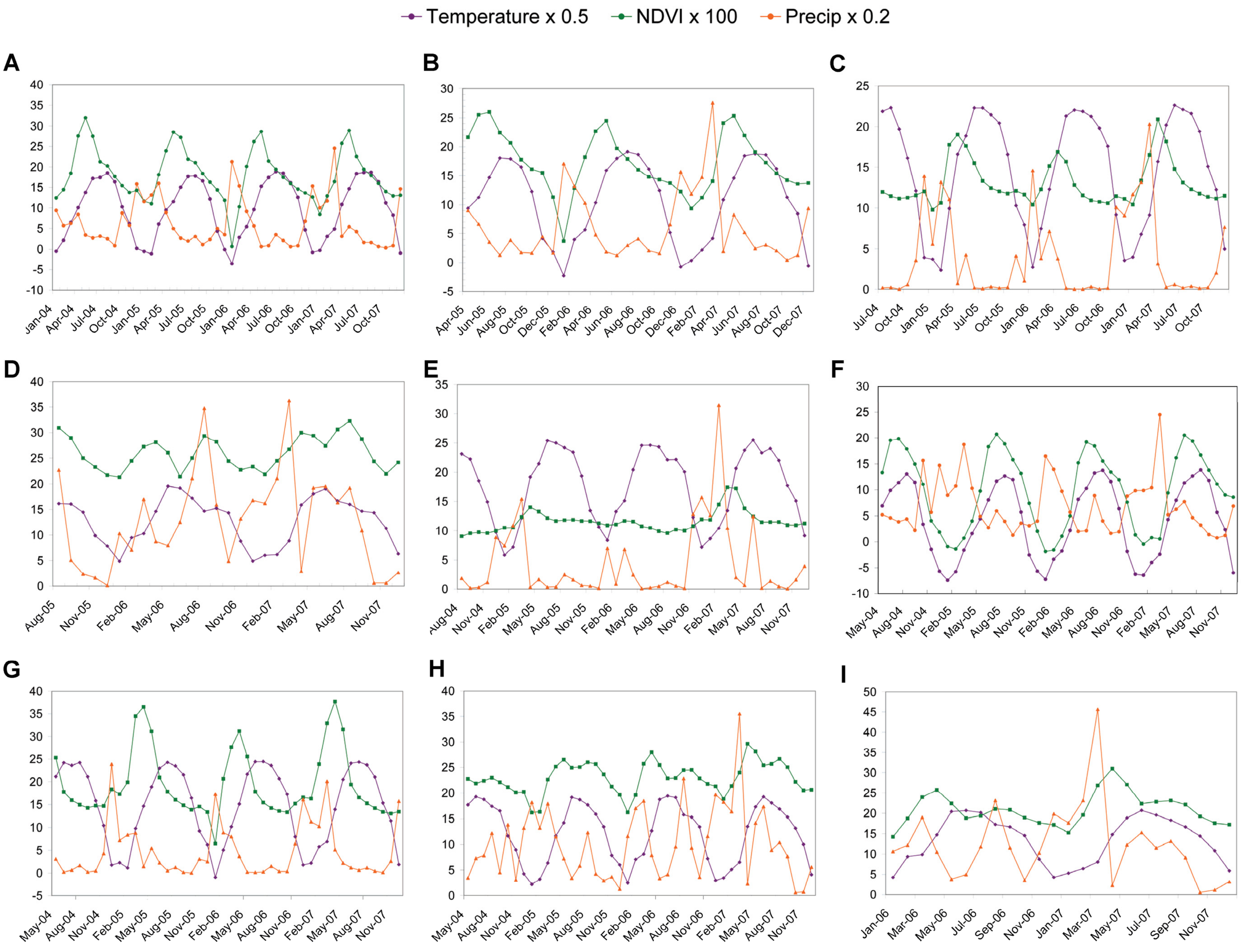

(A) Takhar, (B) Baghlan, (C) Hirat, (D) Khost, (E) Kandahar, (F) Badakhshan, (G) Faryab, (H) Laghman and (I) Nangarhar Province
